# Pancreatic cancer resistance conferred by stellate cells: looking for new preclinical models

**DOI:** 10.1186/s40164-020-00176-0

**Published:** 2020-08-03

**Authors:** Pei Pei Che, Alessandro Gregori, Omidreza Firuzi, Max Dahele, Peter Sminia, Godefridus J. Peters, Elisa Giovannetti

**Affiliations:** 1 Department of Radiation Oncology, Amsterdam University Medical Centers, Location VUMC, Cancer Center Amsterdam, Amsterdam, The Netherlands; 2 Department of Medical Oncology, Amsterdam University Medical Centers, Location VUMC, Cancer Center Amsterdam, CCA Room 1.52 De Boelelaan 1117, 1081 HV Amsterdam, The Netherlands; 3 Medicinal and Natural Products Chemistry Research Center, Shiraz University of Medical Sciences, Shiraz, Iran; 4 Department of Biochemistry, Medical University of Gdansk, Gdańsk, Poland; 5 Cancer Pharmacology Lab, AIRC Start-Up Unit, Fondazione Pisana per la Scienza, Pisa, Italy

**Keywords:** Pancreatic cancer, Therapy resistance, Stellate cells, Deoxycytidine

## Abstract

Pancreatic ductal adenocarcinoma (PDAC) has an extremely poor response to chemo- and (modest-dose conventionally fractionated) radio-therapy. Emerging evidence suggests that pancreatic stellate cells (PSCs) secrete deoxycytidine, which confers resistance to gemcitabine. In particular, deoxycytidine was detected by analysis of metabolites in fractionated media from different mouse PSCs, showing that it caused PDAC cells chemoresistance by reducing the capacity of deoxycytidine kinase (dCK) for gemcitabine phosphorylation. However, data on human models are missing and dCK expression was not associated with clinical efficacy of gemcitabine. We recently established co-culture models of hetero-spheroids including primary human PSCs and PDAC cells showing their importance as a platform to test the effects of cancer- and stroma-targeted drugs. Here, we discuss the limitations of previous studies and the potential use of above-mentioned models to study molecular mechanisms underlying chemo- and radio-resistance.

## To the Editor

We read with great interest the recent article by Dalin and collaborators [1] on liquid chromatography-mass spectrometry analysis and cell viability assays in different models of PDAC cells and PSCs, showing that secretion of deoxycytidine by PSCs conferred resistance to gemcitabine.

**Fig. 1:**
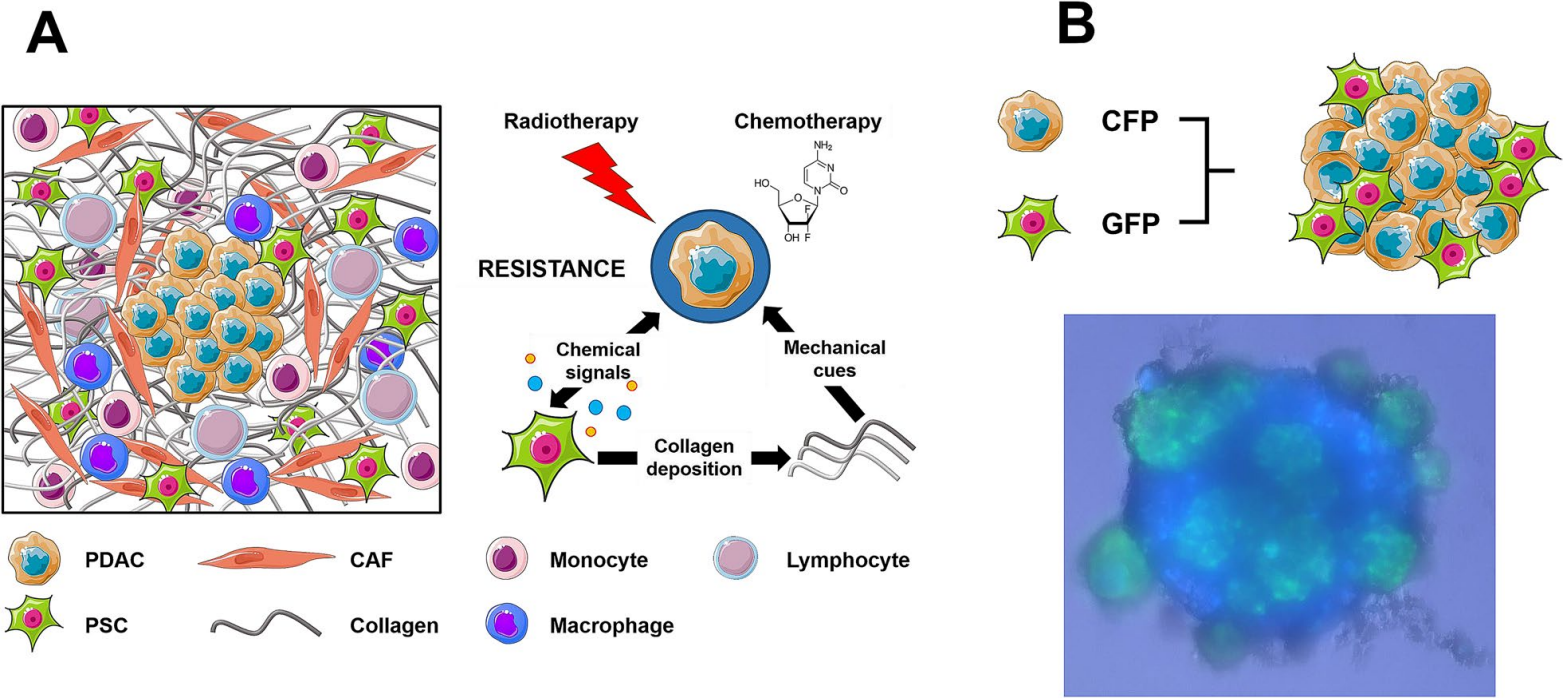
Schematic overview of the surrounding TME in pancreatic cancer. **a** The TME of PDAC is comprised of several components, including CAFs, immune cells and extracellular matrix proteins, like collagen. Activated PSCs and collagen are able to promote resistance against chemo- and radiation treatment via paracrine signaling and mechanical cues, respectively. **b** Representative fluorescent image of co-cultured PSC/PDAC (1:2) spheroid. Green fluorescent protein-expressing PSCs were co-cultured with cyan fluorescent protein-expressing primary PDAC cells. Image was taken 48 h post-seeding. *CAF* Cancer-associated Fibroblasts, *PDAC* Pancreatic Ductal Adenocarcinoma, *PSC* Pancreatic Stellate Cells, *CFP* Cyan Fluorescent Protein, *GFP* Green Fluorescent protein. Some material of this figure was adapted from images made by Servier Medical Art by Servier, licensed under a Creative Commons Attribution 3.0 Unported License, at https://smart.servier.com

PDAC is typically characterized by features associated with poor prognosis: therapy resistance, early metastasis and recurrence [2]. Approximately 80% of PDAC volume is stroma comprising a liquid milieu of cytokines/growth factors and extracellular vesicles, a cellular component (PSCs, fibroblasts, endothelial and immune cells), and an extracellular matrix. These components are interconnected and their communication with cancer cells might affect aggressive behavior and therapy response (Fig. 1a).

Despite inherent and acquired resistance against most chemotherapeutic drugs, cytotoxic chemotherapy remains the mainstay of the treatment for PDAC patients, the majority of whom present with advanced-stage disease. Gemcitabine resistance is multifactorial and PSCs have been implicated in several of the underlying processes [3]. Dalin and collaborators [1] performed an elegant analysis of metabolites in fractionated media from different mouse PSCs, showing that deoxycytidine is present and protects PDAC cells from gemcitabine cytotoxicity, raising the question of whether deoxycytidine affects key oncogenic pathways as well. Notably, mouse macrophages are also able to secrete deoxycytidine [4], but hepatic stellate cells do not. No data are available from primary human PSCs from cancer patients. Primary human PSCs and commonly used PSC-cultures differ both phenotypically and in their interactions with PDAC cells [5], emphasizing the importance of appropriate PSC-PDAC co-culture models, with paired primary cancer cells and PSCs. Moreover, when a PDAC organoid was assessed for the influence of PSC conditioned media, no significant protection against gemcitabine cytotoxicity was observed [1]. This interesting observation suggests that under particular physiological conditions and in 3D models, the microenvironment could behave differently. This motivates efforts to investigate these findings in even more appropriate tumor-TME preclinical models.

We recently established PSC/PDAC spheroids to be used as an important tool for screening of cancer- and stroma-targeted drugs (Fig. 1b). These co-culture spheroids exhibited higher resistance to gemcitabine compared to PDAC-only spheroids, whereas c-MET inhibitors tivantinib, PHA-665752 and crizotinib were equally effective in both spheroid models [6]. The potential of targeting c-MET receptor as a valuable therapeutic strategy in selected cases of PDAC was also shown in a recent in vivo study which demonstrated that a triple combination of gemcitabine with HGF and c-MET inhibitors was the most effective strategy to both reduce the size of primary tumours as well as to completely eliminate metastases [7].

In order to combat the drug- and radioresistance mediated by PSCs in PDAC [8], these novel 3D preclinical model studies are highly preferred to investigate the interaction between pharmacological and radiotherapeutic strategies, for example through testing of combinations of radiosensitizing and cytotoxic agents with radiation. Similar studies have been performed using 3D preclinical models in glioblastoma [9].

Another important question arises concerning the hypothetical role of dCK in gemcitabine resistance. Treatment with PSC media did not reduce intracellular levels of gemcitabine, suggesting no uptake competition, but rescued dCK-catalyzed formation of intracellular deoxycytidine triphosphate (dCTP) levels [1]. It would be of interest to determine the formation of gemcitabine-diphosphate, which inhibits ribonucleotide-reductase, responsible for synthesizing deoxynucleotides required for DNA synthesis/repair. A decreased dCTP would indeed result in reduced feedback inhibition of dCK, and potentiate gemcitabine activation, favoring gemcitabine-triphosphate in its competition with dCTP for incorporation into DNA [10]. Data on the synthesis of gemcitabine-triphosphate and its incorporation into DNA are therefore warranted to further elucidate the mechanism of resistance.

Finally, dCK expression is not clearly associated with clinical efficacy of gemcitabine in PDAC [5]. Further studies on the complex TME comprising distinct cell types, but also hypoxic and stromal dense areas [2], might explain differential effects on pyrimidine metabolism and chemo- as well as radio-resistance.

In conclusion, we look forward to additional studies on optimized preclinical models evaluating the effects of chemotherapy and radiotherapy in PDAC and unravelling the mechanisms behind treatment failure.

## Abbreviations

dCK: Deoxycytidine kinase
dCTP: Deoxcytidine triphosphate
PDAC: Pancreatic ductal adenocarcinoma
PSCs: Pancreatic stellate cells
TME: Tumor microenvironment
